# Distributed education enables distributed economic impact: the economic contribution of the Northern Ontario School of Medicine to communities in Canada

**DOI:** 10.1186/s13561-021-00317-z

**Published:** 2021-06-09

**Authors:** John C. Hogenbirk, David R. Robinson, Roger P. Strasser

**Affiliations:** 1 Centre for Rural and Northern Health Research, Laurentian University, 935 Ramsey Lake Road, Greater Sudbury, Ontario P3E 2C6 Canada; 2 School of Northern and Community Studies, Laurentian University, 935 Ramsey Lake Road, Greater Sudbury, Ontario P3E 2C6 Canada; 3 Professor of Rural Health, and Founding Dean Emeritus, Northern Ontario School of Medicine, 935 Ramsey Lake Road, Greater Sudbury, Ontario P3E 2C6 Canada

**Keywords:** Distributed medical education, Economic impact, Economic contribution, Socio-economic deprivation, Social accountability, Underserviced areas, Ontario, Canada

## Abstract

**Background:**

Medical schools with distributed or regional programs encourage people to live, work, and learn in communities that may be economically challenged. Local spending by the program, staff, teachers, and students has a local economic impact. Although the economic impact of DME has been estimated for nations and sub-national regions, the community-specific impact is often unknown. Communities that contribute to the success of DME have an interest in knowing the local economic impact of this participation. To provide this information, we estimated the economic impact of the Northern Ontario School of Medicine (NOSM) on selected communities in the historically medically underserviced and economically disadvantaged Northern Ontario region.

**Methods:**

Economic impact was estimated by a cash-flow local economic model. Detailed data on program and learner spending were obtained for Northern Ontario communities. We included spending on NOSM’s distributed education and research programs, medical residents’ salary program, the clinical teachers’ reimbursement program, and spending by learners. Economic impact was estimated from total spending in the community adjusted by an economic multiplier based on community population size, industry diversity, and propensity to spend locally. Community employment impact was also estimated.

**Results:**

In 2019, direct program and learner spending in Northern Ontario totalled $64.6 M (million) Canadian Dollars. Approximately 76% ($49.1 M) was spent in the two largest population centres of 122,000 and 165,000 people, with 1–5% ($0.7 M – $3.1 M) spent in communities of 5000–78,000 people. In 2019, total economic impact in Northern Ontario was estimated to be $107 M, with an impact of $38 M and $36 M in the two largest population centres. The remaining $34 M (32%) of the economic impact occurred in smaller communities or within the region. Expressed alternatively as employment impact, the 404 full time equivalent (FTE) positions supported an additional 298 FTE positions in Northern Ontario. NOSM-trained physicians practising in the region added an economic impact of $88 M.

**Conclusions:**

By establishing programs and bringing people to Northern Ontario communities, NOSM added local spending and knowledge-based economic activity to a predominantly resource-based economy. In an economically deprived region, distributed medical education enabled distributed economic impact.

**Supplementary Information:**

The online version contains supplementary material available at 10.1186/s13561-021-00317-z.

## Background

In 2013, medical schools and teaching hospitals had an economic impact in Canada of $66B (billion) CDN (Canadian Dollars) [1]. For distributed medical education (DME) programs, in which academic and clinical programs are offered in communities located away from the main campus, the economic impact is also distributed among participating communities and within the broader region [2, 3]. DME program spending represents new money coming into rural or remote areas, and can help in the economic sustainability of these regions with the potential for improvements in the social determinants of health and health equity, which in turn can have positive economic impact [4–11]. However, with a few exceptions [2, 3], studies typically have been conducted at the level of the province, state, or nation, and while some studies may estimate the impact on capital cities or large regions, the economic impact is not estimated for the smaller cities or towns. Communities have an interest in knowing the community-specific economic impact, given the role of these communities in ensuring the success of DME. Our study sought to fill this information gap to estimate the economic impact of the Northern Ontario School of Medicine’s (NOSM) fully operational community engaged health professional education and research programs for specific communities in the historically underserved and economically disadvantaged region of Northern Ontario.

**Fig. 1:**
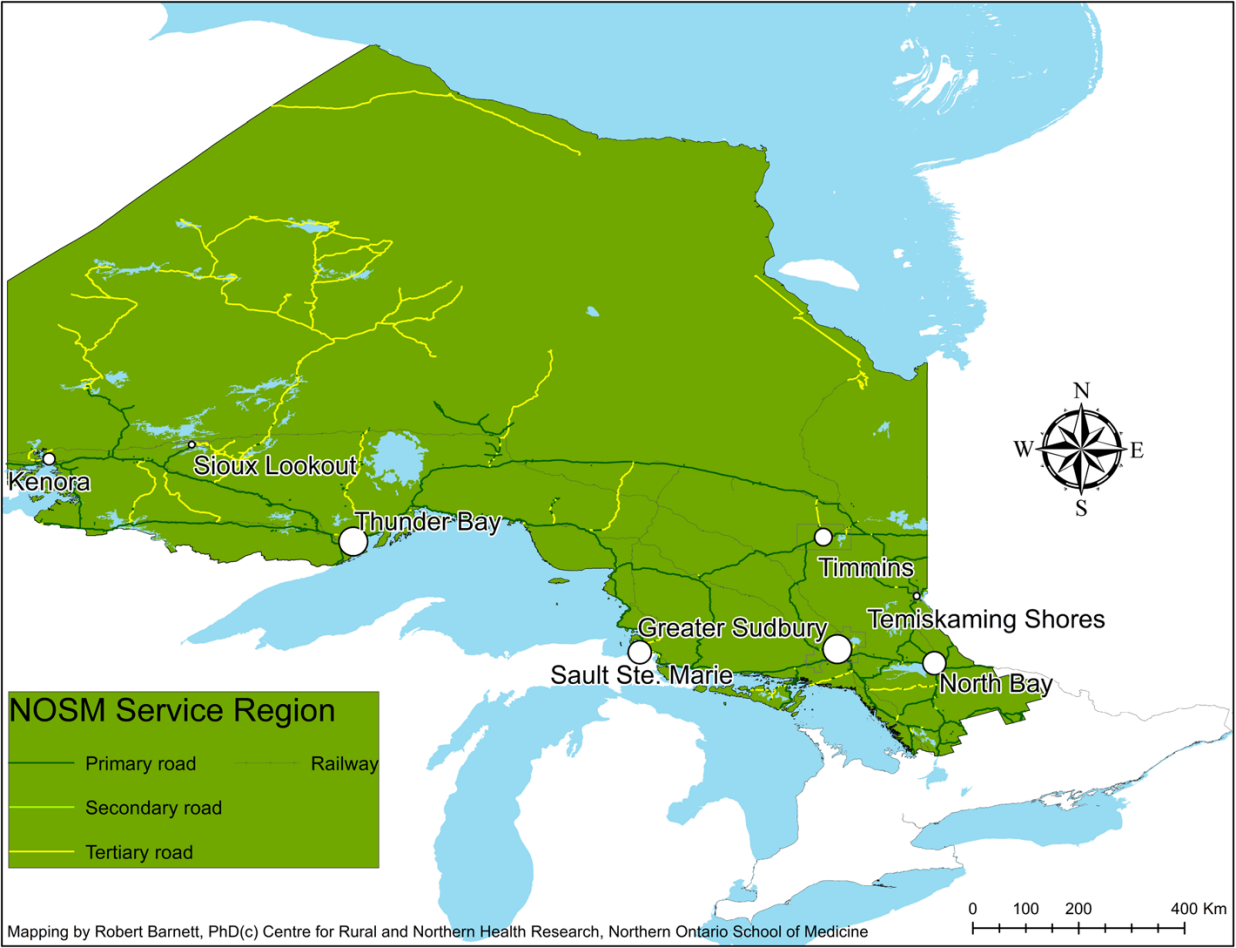
Map showing major communities and transportation network in the service region of the Northern Ontario School of Medicine

NOSM’s service region in Northern Ontario has 90% of Ontario’s land area (806,787 of 908,699 km^2^)—an area that exceeds that of the United Kingdom and France (exclusive of overseas territories)—but has only 6% of the population of the province (840,739 of 13,448,494 people) (Fig. 1) [12]. Communities in the lower part of the service region are connected by road, rail, and air, whereas those communities in the upper part are connected by air and winter (ice) roads. The economy of this region is largely resource based [13], with socio-economic characteristics and population health statuses that are worse than the rest of the province [14]. NOSM’s service region, relative to the whole province, has a higher proportion of Indigenous (14% vs. 2%) and Francophone (24% vs. 5%) people [15–17]. These minority groups have comparatively lower socio-economic status, poorer health status, and worse access to healthcare services [18, 19].

The political decision to locate a stand-alone medical school in Northern Ontario rather than to exploit perceived scale efficiencies of established and larger medical schools in southern Ontario was undertaken to improve overall health outcomes and to counter the high cost of moving patients from remote communities to doctors in the major cities. Previous initiatives aimed at improving access to healthcare services in Northern Ontario were not fully successful [20–22]. NOSM, which started accepting students in 2005, was established with an explicit social accountability mandate to help improve the health of the people of Northern Ontario [23, 24]. The deliberate creation of a new medical school in a historically underserved region sought to leverage the strong positive association between physicians’ practice location and where they spent their childhood [25], as well as the strong positive association between practice location and where physicians completed their medical school education or residency training [22, 26–28]. These training opportunities were extended by NOSM to other healthcare practitioners such as dietitians and rehabilitation therapists. The establishment of NOSM as a distributed medical school was viewed as part of “comprehensive, four-year plan to invest in health and education, foster economic growth and balance the budget” in Northern Ontario [29].

At present, NOSM provides a distributed educational experience in over 90 communities for a broad range of students including undergraduate medical students, postgraduate medical residents, as well as dietetic, rehabilitation therapy, physician assistant, and pharmacy students [24]. In addition, students and graduate students from other health care professional schools undertake placements in the region. These programs, staff, learners, and teachers increase the economic activity in participating communities and surrounding lands. This study sought to estimate the community-specific economic impact of spending attributable to NOSM’s education and research programs and related activities.

## Methods

To estimate the economic impact, we built a cash-flow model using Excel (Microsoft Office Professional Plus 2013, v15.0.5153.1000) for communities clustered in eight economic zones (defined below) and for NOSM’s service region in Northern Ontario in total. To this accounting structure we added a local economic model [30–34] using multipliers that incorporated population size, industry diversity, and the propensity to spend locally—these multipliers were derived from a regression equation developed with data specific to Ontario communities [35, 36].

The eight distinct economic zones included two census metropolitan areas (CMAs, core population ≥ 100,000), four census agglomerations (CAs, core population ≥ 10,000), and two of the larger census subdivisions (CSDs). CMAs and CAs include cities and surrounding lands that represent zones of integrated economic activity as inferred from commuter flows to urban cores [37]. One CSD had been part of a CA in 2001 and 2011 and therefore we grouped spending in all communities that had been part of the former CA, labelling this as Temiskaming Shores CSD+ (CSD plus). The second CSD of Sioux Lookout is a health and social service hub for 29 First Nation (Indigenous) communities distributed across northwest Ontario. NOSM-related spending (described below) in this community was high relative to population size. All other CSDs in the region with NOSM-related spending, including First Nations Indigenous communities, were grouped together to maintain confidentiality. We estimated the economic impact for this group as a whole using a multiplier based on average population size of 3260 people. We also estimated an additional intra-regional economic impact given that community members were known to purchase goods and services from other communities in the region.

Community-specific data that were used to develop the cash-flow model included: salaries and benefits of NOSM personnel and medical residents, and reimbursement for clinical teaching duties; spending on travel, supplies, and services; stipends paid to contract faculty; spending on educational programs; spending on research; and other spending for fiscal year (FY) 2014/2015. These totals include spending recorded through the Paymaster program for salaries of medical residents (one of several learner groups) and the academic Alternate Funding Plan for clinical teachers (Supplement 1). We estimated average local spending per week for all other learners. This average weekly spending was multiplied by the number of learner-weeks per community to estimate annual local spending. We refer to the combined spending on all programs and by all learners as NOSM-related spending. Full postal codes were used to locate the employee, resident, clinical teacher, or vendor in specific communities within NOSM’s service region (Fig. 1).

Cash flow totals were cross-checked against publicly reported values in NOSM’s Financial Statement of Operations [38], with “Amortization” replaced by “Cash flows from financing and investing activities (Obligations and Acquired)” plus payments to residents and clinical teachers. The cash flow model was constructed to best represent actual program, employee, teacher, and learner spending in Northern Ontario communities [39].

Data, particularly spending data, on programs that pre-dated NOSM were not readily obtainable and therefore the counterfactual was the absence of all programs in the service region, reflecting the “gross change in a region’s existing economy that can be attributed to a given industry” [33]. Prior to NOSM, there were no medical school satellite or regional sites in Northern Ontario. Instead, there was a diverse collection of programs affiliated with other Ontario medical schools (Supplement 2). In 2005, NOSM started a new, full 4-year undergraduate medical education program and since then has added five more postgraduate medical specialties; plus physician’s assistant and medical physics programs, and pharmacy placements. In 2005 to 2006, NOSM began consolidation of existing healthcare and medical education programs and has steadily increased enrolment, offered more types of placements, and recruited more healthcare providers, care facilities, and communities into its programs.

The economic model summed direct, indirect, and induced economic effects to estimate the total economic impact of these programs and people in the eight economic zones and for the whole of the service region. The community-specific multipliers combined all effects into a single estimate of economic impact. We used 2016 Canadian census population sizes [12] to calculate the multipliers (described above) that were applied to cash flows to estimate the impact of all monies that were available to be re-spent in the community or region, corrected for monies that leave Northern Ontario.

Detailed spending from FY 2014/2015 was made available to the research team. These spending data were multiplied by the ratio of total spending in FY 2018/2019 divided by total spending in FY 2014/2015 to estimate spending in FY 2018/2019. We checked the assumption that spending patterns were reasonably consistent from year to year by using a Chi-squared test of the count of dollars in each of the 15 major spending categories across five fiscal years. Community-specific multipliers were applied to the adjusted spending. The regional impact was estimated using a multiplier that was 10% higher than the largest community’s multiplier, which seemed reasonable given intra-regional spending. The regional multiplier was applied to total adjusted spending in the region.

We calculated the effect on employment in Northern Ontario to obtain an alternative measure of the economic impact. The number of full time equivalent (FTE) positions included NOSM employees and faculty, as well as employees of health care facilities whose salary and benefits were paid in whole or in part by NOSM, but who were not formally NOSM employees. FTE data also included residents who were also not formally NOSM employees. Data on clinical teachers FTE were not readily available and could not be included. We increased the income multipliers by 4.1% before estimating FTE. This increase was based upon a comparison of income and employment multipliers estimated for census divisions in Northern Ontario [40].

We also calculated a first approximation of the economic impact of NOSM-trained physicians who located their practice in the service region. We used the number of physicians known to be practicing in the service region in November 2018, multiplied by average gross income for family physicians (FPs) in Ontario ($291,090), and adjusted by a published multiplier of 1.07 for family practices in Canada [28, 41, 42]. A regional impact was estimated using a multiplier that was 10% higher, and was applied to total gross income for the region. For simplicity, we assumed that average FP income also applied to other medical and surgical specialists.

## Results

**Fig. 2:**
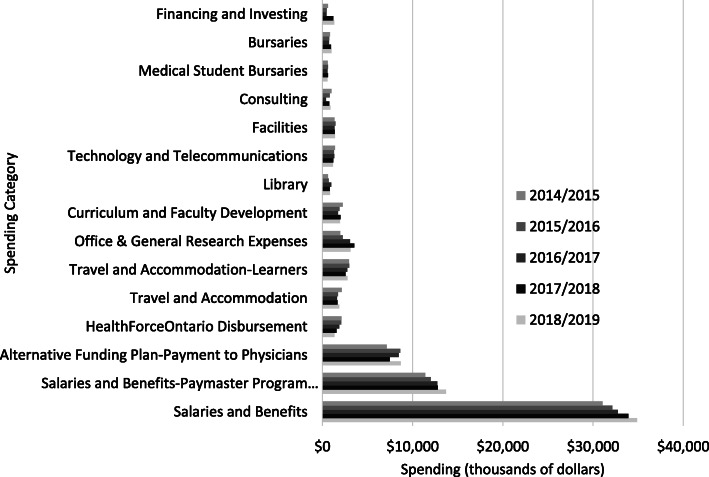
Spending in all geographic zones in fiscal years 2014/2015–2018/2019 by the Northern Ontario School of Medicine and related programs. ^a^ Includes Northern Ontario School of Medicine (NOSM) educational programs and research activities, the Paymaster program for medical resident salaries, and Alternate Funding Plan for clinical teaching reimbursement. Spending by other learners was not included. ^b^ The amounts, counted in the hundreds of thousands of dollars, were not statistically significantly different among 15 major spending categories across all fiscal years (Chi-Squared = 26.4, df = 56, *p* = 1.00)

Adjusted financial statements showed that total spending by NOSM, including salary for medical residents and reimbursement for clinical teaching duties, increased from $37.5 M in FY 2014/2015 to $75.6 M in FY 2018/2019. The amounts, counted in the hundreds of thousands of dollars, were not statistically significantly different among 15 major spending categories across FY 2014/2015 to FY 2018/2019 (Fig. 2) (Chi-Squared = 26.4, df = 56, *p* = 1.00). In FY 2018/2019, an estimated $61.0 M (80.7%) of NOSM’s total spending occurred in the service region, which included $11.2 M in support of programs, $40.8 M for salary of staff and clinical teachers, and $9.0 M for residents’ salary. All other learners were estimated to spend an additional $3.6 M, bringing the estimated grand total spending in the region to $64.6 M in 2019.

**Fig. 3:**
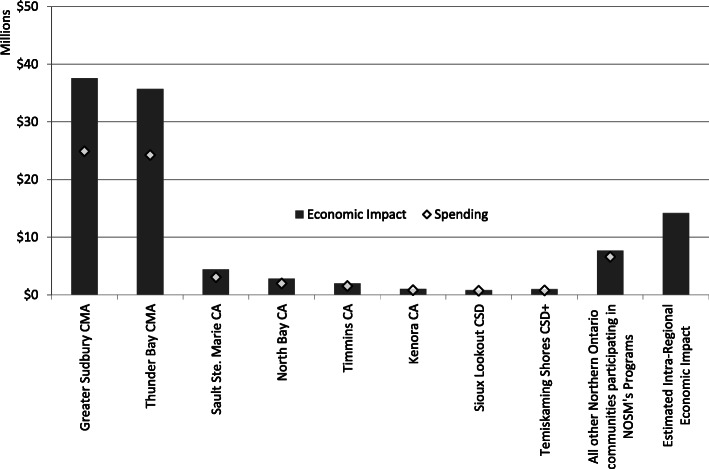
Total spending (◊) and economic impact (bars) (millions of Canadian dollars) in the service region of the Northern Ontario School of Medicine (NOSM) for fiscal year 2018/2019. ^a^ Includes the Northern Ontario School of Medicine (NOSM) educational programs and research activities, the Paymaster program for medical resident salaries, academic Alternate Funding Plan for clinical teaching reimbursement, and spending by other learners. ^b^ CMA: Census Metropolitan Area. ^c^ CA: Census Agglomeration. ^d^ CSD: Census Subdivision. ^e^ CSD+: Census Subdivision, plus all areas that comprised the former CA

The total economic impact in the service region was estimated to be $107 M in 2019 (Fig. 3). This estimate assumed that some of the money that leaked out of one community in Northern Ontario would be spent in another community in Northern Ontario before leaving the region. In the two largest economic zones of Thunder Bay CMA and Greater Sudbury CMA, the economic impact was $38 M (35.0% of total) and $35.7 M (33.3% of total), respectively. The impact of spending in communities outside of these urban areas summed to $19.7 M (18.4% of the total impact). Intra-regional spending contributed an additional $14.2 M (13.3%). Per capita impact generally followed the same pattern, though the Sioux Lookout CSD and the Temiskaming Shores CSD+ had a per capita impact that was surpassed only by Greater Sudbury and Thunder Bay (Table 1).

**Table 1:** Per capita and employment impact of the Northern Ontario School of Medicine and related programs in Northern Ontario ^a^

	Greater Sudbury CMA ^**b**^	Thunder Bay CMA	Sault Ste. Marie CA ^**c**^	North Bay CA	Timmins CA	Kenora CA	Sioux Lookout CSD ^**d**^	Temiskaming Shores CSD+ ^**e**^	All other participating Northern Ontario communities	Intra-regional economic impact ^**f**^	Total for Northern Ontario
2016 Census Population	164,689	121,621	78,159	70,378	41,788	15,096	5272	12,940	257,802 (average 3263)	–	**859,994**
Income Multiplier [35, 36]	1.51	1.47	1.44	1.43	1.28	1.27	1.17	1.27	1.16	–	**1.66**
Per capita impact	$228	$294	$56	$40	$47	$68	$155	$75	$30	–	**$125**
Employment Impact Full Time Equivalents	264	248	21	23	7	5	6	5	42	81	**702**
Number of NOSM-trained physicians practising in community ^g^	57	59	30	26	20	5	9	3	47 ^h^	–	**256**
Economic impact of physicians (millions)	$17.8	$18.4	$9.3	$8.1	$6.2	$1.6	$2.8	$0.9	$14.6 ^h^	$8.0	**$87.7**

^a^Northern Ontario School of Medicine (NOSM) educational programs and research activities, the Paymaster program for medical resident salaries, the academic Alternate Funding Plan for clinical teaching reimbursement, and spending by learners

^b^*CMA* Census Metropolitan Area

^c^*CA* Census Agglomeration

^d^*CSD* Census Subdivision

^e^*CSD+* Census Subdivision plus all areas that comprised the former CA

^f^Impact was calculated as the difference between the total for the region and sum for participating communities. Population size, multiplier, and per capita impact were not applicable

^g^Included family physicians and other medical or surgical specialists who completed undergraduate or postgraduate medical education or both at NOSM and had been in fully qualified practice for at least one year as of November 2018

^h^This value included all other communities in the service region with NOSM-trained physicians, regardless of whether the communities participated in NOSM programs

Spending in 16 First Nations (Indigenous) communities averaged $8900 with an estimated impact of $10,400 per community. This represented a per capita impact of $10 in communities that ranged in size from approximately 140 to approximately 2500 people (average of 1012 people).

There were 404 full time equivalent (FTE) positions in 2019 in Northern Ontario with an employment impact of 702 FTEs in the region (Table 1). The pattern of employment impact in the region mirrored that of income impact.

In November 2018, there were 226 family physicians and 30 other medical or surgical specialists who had trained at NOSM and were practising in the region (Table 1). The economic impact of these physicians in the region was estimated to total $87.7 M.

## Discussion

For every dollar spent by NOSM, including monies spent in support of clinical duties by residents, reimbursement for teaching duties by physicians, and spending by learners, an estimated $0.66 was generated in additional economic activity in 2019 in NOSM’s service region of Northern Ontario. Although 68% of economic impact occurred in the two largest population centres, other cities and towns in the region shared 18% of the economic impact, while the intra-regional economic impact was estimated at 13%. The economic impact in Northern Ontario increased by 60% over eleven years, from $67 M in 2008 [2] to $107 M in 2019, which reflected NOSM’s much expanded suite of education programs, research activities, additional learners, and other funding that flowed through NOSM. If the same multipliers are used in both studies, then NOSM’s economic impact increased by 94% to $130 M.

There are only a few published studies that have examined the economic impact of distributed medical education programs on individual communities. In 2010, the economic impact of Montana’s part of the Washington, Wyoming, Alaska, Montana and Idaho (WWAMI) medical education program in clinical teaching sites located away from the main campus in Bozeman was estimated to be $7.2 M USD [3], equivalent to $8.7 M CDN in 2019. In comparison, the total annual economic impact of NOSM in 2019 was $34 M outside of the two largest population centres: four times that of Montana’s WWAMI program.

A local comparison comes from an economic impact study of the academic health sciences centre in Greater Sudbury (Health Sciences North-HSN) [35]. HSN had an economic impact of $310 M in the city and $2.6 M in nearby communities in FY 2010/2011—equivalent to $345 M and $2.9 M in FY 2018/2019. Although HSN revenues and expenditures were 8-times higher than that of NOSM, HSN’s impact outside of Sudbury was one-twelfth of NOSM’s impact outside of Sudbury or Thunder Bay. Much of this difference can be explained by a difference in mandates and organizational structure. For instance, HSN serves as the hospital for Greater Sudbury as well as a tertiary and quaternary care referral centre for northeast Ontario, with each community having its own independent hospital. In comparison, NOSM has central campuses in Greater Sudbury and Thunder Bay, with teaching sites in over 90 communities across northeast and northwest Ontario. Notwithstanding the differences in organizational mandates, NOSM’s 12-fold higher impact outside of the major urban areas demonstrated a distributed impact.

However, the economic impact relative to the gross domestic product (GDP) of the region was small. The best available information suggested that the economic impact of $107 million represented 0.3% of the region’s GDP [43, 44]. It is also important to note that spending and economic impact disproportionately accrued to the larger population centres of Greater Sudbury and Thunder Bay as evidence by the higher per capita impact values. More could be done to achieve an equitable distribution while recognizing differences in infrastructure, industry diversity, population size, proximity to larger centres, propensity to spend locally, and other salient economic characteristics as well as pertinent programmatic opportunities and challenges.

Regardless of the proportion of GDP and per capita impact, spending by DME programs in participating communities and the impact associated with re-spending constitutes an investment in economically deprived regions and may help improve employment, income, education, and other social determinants of health [8, 45, 46]. In many communities, this spending represents new money. Findings from an earlier study [2, 47], from a similar study conducted on a DME program in Québec [48], and a study that specifically examined impact on recruitment in DME communities [49] have demonstrated additional social and economic benefits in participating communities. These studies have also shown an increase in civic pride, reputation, networking opportunities, recruitment of healthcare professionals, attractiveness to new businesses, and other benefits in the community. There was more than dollars at work, though new dollars helped.

### Limitations

There are practical and theoretical limits to local economic impact analyses [30–34]. Nonetheless, this approach is considered reasonable for short-term estimates in small, simple economies [31] such as Northern Ontario and it is commonly used to estimate the economic impact of universities, teaching hospitals, and medical schools [1, 3, 30].

The counterfactual was the absence of any of the programs and activities associated with NOSM. This was used because of the difficulty in obtaining program spending information before NOSM, and because NOSM subsumed all previous programs, added more programs, and increased the number of learners, staff, and teachers. Consequently the net economic impact of NOSM may be lower than estimated by our model. However, our model did not measure all benefits (described later in the discussion), which may justify the higher estimate.

In the absence of detailed spending data for 2019, the model used a ratio to adjust spending in 2015 to that in 2019. An examination of spending in broad categories showed no significant differences across five fiscal years and so the use of this ratio seemed reasonable.

Income multipliers were developed prior to 2012 for communities in Ontario and do not differentiate among spending type. Community population size is the sole independent variable, though the formula accounted for industry diversity and propensity to spend locally [36]. Nonetheless, these multipliers were in the range estimated in 2019 for the health care and social assistance sector in Northern Ontario [40]. Increasing the income multiplier by 4% to estimate employment impact seemed reasonable given a similar difference between income and employment multipliers in the aforementioned publication [40].

### Unmeasured benefits

Our approach did not consider all economic activity linked to NOSM. For instance, the model excluded some spending by graduate research students, visitor spending, and construction costs—all three of which were minimal. With a focus on NOSM programs and activities, the model included spending of funds that reimbursed clinicians for teaching duties, but not other types of clinician spending. This additional economic impact can be large [49, 50]. For example, a very preliminary estimate suggested that NOSM-trained physicians who located their practice in the region had an economic impact of $88 M.

Also out-of-scope was any change in the economic burden associated with improved health status or social impact [47, 51] attributable to NOSM. We expect that these benefits have accrued, but we do not have evidence to support this claim. On the other side of the equation, the model excluded the cost of municipal services required by NOSM employees, learners, or clinical teachers. However, these demand costs may be negligible or negative, given that the population is stable or declining in most Northern Ontario communities [12].

Our study did not assess how the economic impact of NOSM-related spending compared to other existing or potential provincial healthcare initiatives. The timing and focus of new government project and program expenditures is complex and largely opaque, but there is no reason to think that NOSM displaced other public spending for healthcare or development in Northern Ontario. On the contrary, it is likely that the presence of NOSM has attracted other developments in academic and health sectors including the health research institutes in Thunder Bay and Sudbury. Nor is there any reason to think that NOSM displaced monies that were otherwise going to frontline care in the region. It is possible that NOSM reduced the need to transport some patients to large centres for primary or ambulatory care, but this is probably a small effect. Future study is required to account for all costs and benefits to assess the relative impact on economic activity.

## Conclusions

Our economic impact study demonstrated that NOSM’s DME programs and associated activities, spending by staff, clinical teachers and learners, and research activities contributed to the Northern Ontario economy in a way that extended beyond the production of health care professionals. In Northern Ontario, the economic impact on participating communities was at least 60% greater than the original government investment. This expenditure in a low resource region provided an economic stimulus and, along with NOSM graduates who set up practice in the region, may help improve the social determinants of health and the health of the population. DME is also DEI—distributed economic impact.

## Supplementary Information

**Additional file 1.** Explanation of Spending Envelopes.

**Additional file 2.** Educational Programs before and after the inception of the Northern Ontario School of Medicine.

## Data Availability

Aggregated data are available from the corresponding author upon reasonable request.

## Abbreviations

CA: Census agglomeration (www12.statcan.gc.ca/census-recensement/2016/ref/dict/geo009-eng.cfm)
CDN: Canadian
CMA: Census metropolitan area (www12.statcan.gc.ca/census-recensement/2016/ref/dict/geo009-eng.cfm)
CRaNHR: Centre for Rural and Northern Health Research-Laurentian (www.cranhr.ca)
CSD: Census subdivision (www12.statcan.gc.ca/census-recensement/2016/ref/dict/geo012-eng.cfm)
DEI: Distributed economic impact
DME: Distributed medical education
FP: Family (medicine) physicians/practitioners
FTE: Full time equivalent
FY: Fiscal year
HSN: Health Sciences North (www.hsnsudbury.ca)
NOSM: Northern Ontario School of Medicine (www.nosm.ca)
