# Supplementary material for: Distributed education enables distributed economic impact: the economic contribution of the Northern Ontario School of Medicine to communities in Canada
Source: Health Econ Rev. 2021 Jun 9;11:20. doi: 10.1186/s13561-021-00317-z (PMC8191106; doi:10.1186/s13561-021-00317-z)
Supplement: Supplementary file 1 — Additional file 1. Explanation of Spending Envelopes. [file 13561_2021_317_MOESM1_ESM.docx]

Supplement 1: Explanation of Spending Envelopes

Hogenbirk et al. Distributed education enables distributed economic impact: the economic contribution of the Northern Ontario School of Medicine to communities in Canada.

The total economic activity included: spending on **Northern Ontario School of Medicine** (NOSM) educational programs and research activities; spending on the **Paymaster** and academic **Alternate Funding Plan** (AFP) programs; plus spending by **learners**.

**Northern Ontario School of Medicine (NOSM)**: This included spending on educational programs and research activities. New educational programs comprised the undergraduate medical education, dietetic internship, rehabilitation therapist, physician assistant, and pharmacist programs. Expanded and redesigned programs included placements for student and graduates of other medical schools and postgraduate (residency) medical education programs. Spending on research activities included grant or scholarship support of Masters and PhD candidates. All spending on educational programs and research activities was included.

**Paymaster (medical residents)**: This program provides salary and benefits to medical residents in Ontario for patient services that residents provide to hospitals and other health care facilities. NOSM administers these monies to residents at NOSM on behalf of the Professional Association of Residents of Ontario (PARO, <http://www.myparo.ca/>). PARO negotiates the contract with the Council of Academic Hospitals of Ontario on behalf of medical residents. This spending was included in the calculation of economic impact.

**Alternate Funding Plan (AFP) (clinical preceptors)**: The Northern Ontario Academic Medicine Association (NOAMA, <http://www.noama.ca>), manages the academic Alternate Funding Plan (AFP) on behalf of the Ontario Ministry of Health and Long‐Term Care and NOAMA members, which includes the Physician Clinical Teachers’ Association (<http://www.noama.ca/site/pcta/>). The AFP reimburses physicians for their time engaged in teaching medical learners. Physicians are organized into Local Education Groups (LEGs) to facilitate the provision of distributed research and teaching services in Northern Ontario (<http://localeducationgroups.ca/>). The funds flow through NOSM and were included in the spending totals and estimated economic impact.

**Learners**: Learners included: medical students; medical residents; dietetic, rehabilitation therapy; physician assistant; and pharmacy students. These learners may have been enrolled at NOSM or at other health and medical education schools in Canada or internationally and participated in NOSM-administered placements in the region. Detailed information on the number of learners and the duration of their stay in each community were averaged over three fiscal years (2013/2014 to 2016/2017 or 2014/2015 to 2016/2017 for pharmacy students) and used to estimate learner spending in these communities. Estimated learner spending was based on spending calculated for undergraduate students enrolled at Laurentian University in 2008 [1], and adjusted by inflation. NOSM personnel familiar with the different medical and health learner programs were consulted to ensure that all spending, whether by NOSM or by the learner, was included in the model and that no spending was double counted.

Reference for Supplement

1. Robinson D. 2008. Economic Impact of the Northern Ontario School of Architecture (NOSOA) V3. INORD Working Paper #5-08. Prepared for the NOSOA Steering Committee. The Institute for Northern Ontario Research and Development. Downloaded July 6, 2020 from: <http://inord.laurentian.ca/6_08/Economic_Impact_Northern_Ontario_School_of_Architecture.pdf>.
