# Supplementary material for: Distributed education enables distributed economic impact: the economic contribution of the Northern Ontario School of Medicine to communities in Canada
Source: Health Econ Rev. 2021 Jun 9;11:20. doi: 10.1186/s13561-021-00317-z (PMC8191106; doi:10.1186/s13561-021-00317-z)
Supplement: Supplementary file 2 — Additional file 2. Educational Programs before and after the inception of the Northern Ontario School of Medicine. [file 13561_2021_317_MOESM2_ESM.docx]

**Supplement 2:** Educational Programs before and after the inception of the Northern Ontario School of Medicine

Hogenbirk et al. Distributed education enables distributed economic impact: the economic contribution of the Northern Ontario School of Medicine to communities in Canada.

| **Pre-NOSM** | **NOSM** |
| --- | --- |
| **Medical Doctor (MD) Program** | |
| *There was no full length MD program in Northern Ontario prior to NOSM.* | **The MD program** started in 2005/2006. NOSM students spend 40% of their time studying in Indigenous, small and mid-sized urban Northern communities with the remaining time in Greater Sudbury and Thunder Bay.  **Duration:** 4 years  **Enrolment:** 56 per year in 2005/2006; increased to 64 in 2010/2011. Currently 256 students in UG1 to UG4.  **Communities:** 70 in 2005/2006. Over 90 across Northern Ontario in 2018/2019. |
| **Postgraduate Family Medicine Specialty Residency Training Program** | |
| 1. **Northeastern Ontario Family Medicine Program (NOFM;** administered by the Northern Ontario Medical Education Corporation (NOMEC; started in 1991 as a two-year certified family medicine residency program affiliated with the University of Ottawa. It offered clinical learning placements in community-based family medicine leading to certification by the College of Family Physicians of Canada. 2. **Duration:** 2 years   **Enrolment:** 5 residents in 1991; increasing to 16 by 2005.   1. **Communities:** 5 in NE Ontario in 2005 | *Administrative responsibility was transferred to NOSM in 2006 and the NOSM accredited program began in 2007.*  **NOSM Family Medicine** is a accredited two-year residency in Family Medicine. It offers clinical learning in community-based family medicine leading to certification by the College of Family Physicians of Canada. Residents, assigned to individual preceptors, are provided with community-based family medicine rotations in a variety of culturally diverse communities at sites throughout Northern Ontario.  **Duration:** 2 years  **Enrolment:** 30 in 2006; 83 in 2018.  **Communities:** Initially there were 5 training sites: Sudbury; Thunder Bay; North Bay; Sault Ste Marie; and Timmins, plus 21 other communities in Northern Ontario.  The program underwent some restructuring and realignment over the intervening years—this restructuring is not described here.  In 2018/2019, residents were assigned to one of 18 home bases, with placements in at least 30 additional communities across Northern Ontario. |
| 1. **Family Medicine North (FMN) program**, administered by the Northern Ontario Medical Program (NOMP), started in 1990, was a full two-year academic and clinical family medicine residence program based solely in the north and affiliated with McMaster University. The program offered clinical learning in community-based family medicine leading to certification by the College of Family Physicians of Canada. 2. **Duration:** 2 years   **Enrolment**: 5 residents in 1991, increasing to 12 and then 16 per year by 2005.  **Communities:** 13 in NW Ontario in 2005. |  |
| **Postgraduate Family Medicine PGY3 Residency Training Program** | |
| **Northeastern Ontario Family Medicine - PGY3 Advanced Skills Program and NE Ontario Re-entry Program** (administered by NOMEC) started in 2001 provided 3^rd^ year family medicine residencies in Emergency Medicine; Anesthesia; Maternity Care; Palliative Care (in collaboration with University of Ottawa; and Care of Elderly (in collaboration with University of Ottawa).  **Duration:** 1 year  **Enrolment:** 1 in 1991; increasing to 13 from 2001-2006.  **Communities:** 5 in NE Ontario in 2005. | *NOSM consolidated these program beginning in 2007.*  **Family Medicine PGY3 Program**, provides additional learning opportunities that enhance family physicians’ scope of practice and further develop competencies and skills sets. Programs include: Family Medicine Emergency Medicine, Family Practice Anesthesia, Care of the Elderly, Obstetrical Surgical Skills, and Self Directed Enhanced Skills.  **Duration:** 1 year  **Enrolment:** 14 residents in 2009; 18 in 2013; 16 in 2018  **Communities:** 5 in 2005, based primarily in the 6 larger communities in 2018 |
| **Northwestern Ontario Specialty - PGY3 Program** (administered by NOMP) started in 2002 in partnership with McMaster University. Provided a 3^rd^ year of training in Community Internal Medicine and Community General Surgery.  **Duration:** 1 year  **Enrolment:** 2 residents  **Communities:** Thunder Bay as primary site, with placements in 4 communities in NW Ontario. |  |
| **Royal College Specialty Residency Training Program** | |
| **Northeastern Ontario Stream Residency (NOSR) Program** (administered by NOMEC) started in 2002, comprehensive training in Internal Medicine and General Surgery accredited through the University of Ottawa.  **Duration:** 5 years  **Enrolment:** 3 residents  **Communities:** 5 communities in NE Ontario. | *NOSM consolidated and expanded these program beginning in 2007.*  **Royal College Specialty Training Programs** provide training in seven specialties: Anesthesiology; General Surgery; Internal Medicine; Orthopaedic Surgery; Paediatrics; Psychiatry; plus Public Health and Preventative Medicine.  **Duration:** 4 or 5 years.  **Enrolment:** 18 in 2006; 80 in 2013; 84 in 2018.  **Communities:** 15 in Northern Ontario in 2009. 22 in Northern Ontario in 2018. Some rotations are provided at sites outside of Northern Ontario. |
| **Northwestern Ontario Medical Program Community Specialty Programs** (administered by NOMP) started in 2002, comprehensive training in Internal Medicine General Surgery, in affiliation with McMaster University.  **Duration:** 5 years  **Enrolment:** 4 residents.  **Communities:** Thunder Bay as primary site, with placements in 4 communities in NW Ontario. |  |
| **Undergraduate/Postgraduate Electives and Clinical Rotations** | |
| **NE Ontario Electives (NEP) Program** was developed by NOMEC in 1995 with the **NE Ontario Postgraduate Specialty (NOPS) Program** added in 2000, both affiliated with University of Ottawa. Offered rural and remote clinical learning core rotations and electives for undergraduates and postgraduate residents in Family Medicine and other specialties.  **Duration:** average 12 weeks  **Enrolment:** 120 undergraduate and 65 postgraduate students per year. NOPS: total of 63 residents during 2000-2005  **Communities:** 12 communities in NE Ontario. | *NOSM consolidated these program beginning in 2007.*  **The Northern Electives Program (NEP)** provides a variety of high quality rural, remote and small urban clinical learning opportunities in Northern Ontario. It is designed for undergraduate medical students and postgraduate residents from both family medicine and specialty programs. Medical trainees from all Canadian and international Faculties of Medicine are eligible.  **Duration:** 2-8 weeks  **Enrolment:** 224 in 2007  **Communities:** 33 in 2008; 90 in 2018. |
| **Northwestern Ontario Medical Programme (NOMP)** was established in 1972 as partnership between Thunder Bay Medical Society, Northwestern Ontario Medical Society and McMaster University. Community-based elective and core rotations were provided for undergraduates and postgraduates in Family Medicine and other Specialties.  **Duration:** 4 weeks  **Enrolment:** 125 placements.  **Communities:** 22 communities in NW Ontario. |  |
| **Undergraduate /Summer Studentships/Medicine and Allied Health Professionals** | |
| **Northern Academic Health Sciences Network (NAHSN) – Summer Student Studentship Program** **(SSP)** –Originally started as pilot project in 1982 at Health Sciences North in Thunder Bay as OT/PT summer learning experience for McMaster Students. The program was delivered by Health Sciences North (*see note 1*) from 1990-1998. In 1999, the program was integrated into NAHSN and expanded to include learners from 24 regulated health profession programs. While geared for northern students, southern students could apply for studentships and were considered if there were vacancies.  **Duration:** 8 weeks  **Enrolment:** 12 in 1990; increasing to 45 in 2001  **Communities:** In 2008: 6 communities in NE Ontario and 8 in NW Ontario. | **Northern Ontario Summer Studentship Program (SSP)** gives priority to health professional students of any school in Ontario who come from Northern Ontario. The SSP provides paid jobs in health care settings. The work is varied and may include research, special projects or “shadowing”. The intent of the SSP is to enhance the learners’ knowledge of healthcare in the North.  **Duration:** 4-8 weeks  **Enrolment:** 75 in 2008  **Communities:** 18 in 2008. |
| **Rehabilitation Studies** | |
| **Rehabilitation Studies - Northern Studies Stream** – began 1990 in Thunder Bay at Health Sciences North as a northern studies stream for graduate OT/PT learners. Provided training required for northern and rural practice, including knowledge of determinants of health, First Nations concerns and unique skills required for rural practice. In 1999, the program was affiliated with the Rehabilitation Studies Stream of NAHSN.  **Duration:** 14 weeks  **Enrolment:** 18 learners per year  **Communities:** 8 communities in NW Ontario. | **The Northern Studies Stream (NSS)** program, created in 1990 continues “as an integral part of McMaster University’s Physiotherapy, Occupational Therapy and Speech-Language Pathology programs.” Open to McMaster students.  “The **Rehabilitation Studies Stream (RSS)** program allows learners from Rehabilitation Science programs at the University of Toronto, University of Ottawa, Queen’s University and Western University to come to Northern Ontario to complete clinical placements that are part of their respective programs.”  “Learners from audiology, occupational therapy, physiotherapy and speech-language pathology programs at partnering universities apply through their home program to complete a clinical placement in Northern Ontario.”  **Duration:** 6 weeks in northern communities.  **Enrolment:** 198 in 2008; 216 in 2013.  **Communities:** 22 in 2008; 22 in 2018. |
| **NW Ontario - Rehabilitation Studies Stream (NAHSN) Program** started in 1999. The program is offered to Rehabilitation Sciences students in Physiotherapy, Occupational Therapy, Speech and Language Pathology and Audiology.  **Duration:** 6 weeks  **Enrolment:** 30 in 1999; increasing to 51 in 2005.  **Communities:** 9 communities in NE Ontario. |  |
| **Dietetic Internship Program** | |
| **Health Canada First Nations and Inuit Health (FNHIB) Dietetic Internship Program** started in 2002, in partnership with Dietetics Association of Canada, to place interns in urban, rural and remote hospitals, nursing homes, aboriginal health access centres, community health centres, public health units and clinics.  **Duration**: 42 weeks  **Enrolment:** 4-5 students per year  **Communities**: Sudbury and Thunder Bay, with regional placements in communities in NE and NW Ontario. | **Northern Ontario Dietetic Internship Program** started in 2007, it is a pan-Northern initiative with multiple facilitators, sites and facilities throughout Northern Ontario. Open to graduates of accredited dietetic programs who wish to become registered dietitians.  **Duration:** 46 weeks (a minimum of 4-6 weeks in northern communities)  **Enrolment:** 12 in 2009. 12 in 2018.  **Communities:** 10 in 2008; |
| **Interprofessional Continuing Education** | |
| **The Northern Outreach Program** (NOP) was initiated in 1980 by the Health Sciences Faculties at the University of Western Ontario. Purpose involved enriching health services offered by professionals practicing in Northern Ontario. The program supported education, research and additional supports around Communicative Disorders, Library Science, Nursing, Occupational Therapy and Physical Therapy.  **Duration**: 150 sessions per year, delivered on-site and via teleconferencing  **Enrolment:** Average of 550 learners per year  **Communities:** across NW Ontario. | **Lakehead University and NOSM** established Northern Interprofessional Centre for Health Education (NICHE) in 2008.  **Duration:** Ongoing, weekly tutorial sessions held with peers  **Enrolment:** Variable  **Communities:** All Northern communities with practicing health professionals; southern Ontario sites may host sessions. |
| **Health Sciences North (HSN)** – **Interprofessional Education** – began 1991 in Thunder Bay. Offered case-based small group interprofessional continuing professional development, with focus on enhanced cultural sensitivity and increased knowledge and understanding of population demographics and health problems encountered by professionals in NW Ontario. Also supported communities of practice, tele-learning CPD events, learning partnerships (OT,PT, SLP/Audiology) and Practice-Based Research tutorials. Supports included the Northern Ontario Virtual Library (NOVL).  **Duration:** Delivered through North Network tele-learning and web-streaming system.  **Enrolment:** Varying  **Communities:** across Northern Ontario. |  |
| **Other Programs** | |
| *There were no physicians assistant, program medical physics program, or pharmacy placements in Northern Ontario prior to NOSM.* | **Physician Assistant**, started in 2010, the Bachelor of Science Physician Assistant degree is a University of Toronto degree delivered in collaboration with Northern Ontario School of Medicine and The Michener Institute of Education.  **Duration:** 20 weeks  **Enrolment:** 15 in 2013;  **Communities:** |
|  | **Medical physics residency**, started in 2016, open to those with Master’s degree or PhD in medical physics.  **Duration:** 2 years  **Enrolment:** 2 graduates and 2 incumbents since December 2016.  **Communities:** currently offered in Greater Sudbury and Thunder Bay, the two communities with radiation treatment centres. |
|  | **Pharmacy student placements**, started in 2014, open to students in the School of Pharmacy, University of Waterloo.  **Duration:** 6 months  **Enrolment:** 384 students 2015-2017  **Communities:** 4 in 2017. |
| **Sources** | |
| Major sources of information about pre-NOSM programs:  Northern Ontario Medical Education Corporation (NOMEC). A Partner with the Northwestern Ontario Program (NOMP) in the Northern Academic Health Science Network (NAHSN). May 2001 Report.  McCready, W., J. Jamieson, M. Tran and S. Berry. The first 25 years of the NW Ontario Medical Program. *Canadian Journal of Rural Medicine.* 2004; 9(2): 94-100.  NOMP newsletters, brochures and other publications from Health Sciences North, Thunder Bay.  Personal communication with Sue Berry, Miriam MacDonald, and Jeanette Salmi (NOMEC, NOSM).  <http://aix1.uottawa.ca/~fammed/nomhist.htm>  Tepper J, Schultz S, Rothwell D, Chan B. Physician services in rural and northern Ontario. ICES investigative report. Toronto, ON: Institute for Clinical Evaluative Sciences; 2006. | Major sources of information about NOSM programs:  NOSM web-site: [www.nosm.ca](http://www.nosm.ca); https://www.nosm.ca/about/about-nosm/nosm-facts/  Association of Faculties of Medicine of Canada, Canadian Medical Education Statistics (AFMC, CMES): <https://www.afmc.ca/sites/default/files/pdf/CMES/CMES2018-Complete_EN.pdf>  Canadian Post-M.D. Education Registry (CAPER): <https://caper.ca/sites/default/files/pdf/province/2018-ON-SectionB.pdf>  Information on pharmacy placements: <https://uwaterloo.ca/pharmacy/pharmd-program-overview> |
| *Note 1: Health Sciences North was an academic unit at Lakehead University in Thunder Bay, last appearing in the 2011/2012 university calendar (*[*http://csdc.lakeheadu.ca/Catalog/previouscals/2011-2012/pg57.html*](http://csdc.lakeheadu.ca/Catalog/previouscals/2011-2012/pg57.html)*).*  *Health Sciences North (HSN) is also the name of the regional hospital in Greater Sudbury having changed its name to HSN in 2012 (*[*https://en.wikipedia.org/wiki/Health_Sciences_North*](https://en.wikipedia.org/wiki/Health_Sciences_North)*).* | |
